# Granular Cell Ameloblastomatous Transformation From the Remnants of a Dentigerous Cyst: A Unique Case Report

**DOI:** 10.7759/cureus.85021

**Published:** 2025-05-29

**Authors:** Manjula Hebbale, Prashant Rao, Rajshekhar Halli, Priya Deo

**Affiliations:** 1 Oral Medicine and Radiology, Bharati Vidyapeeth (Deemed to be University) Dental College and Hospital, Pune, IND; 2 Oral Pathology and Microbiology, Bharati Vidyapeeth (Deemed to be University) Dental College and Hospital, Pune, IND; 3 Oral and Maxillofacial Surgery, Bharati Vidyapeeth (Deemed to be University) Dental College and Hospital, Pune, IND

**Keywords:** dentigerous cyst, enucleation, granular cell ameloblastoma, lysosomal aggregation, neoplasm

## Abstract

Granular cell ameloblastoma is a unique, infrequent histologic variant of unicystic/multicystic ameloblastoma showing distinct histologic and immunohistochemical features. The prognosis and treatment are similar to other common subtypes of solid or multicystic ameloblastoma. Granular cell ameloblastoma should be distinguished from other lesions with granular cells mainly due to its high risk of recurrence. Although it is rare, it has greater recurrence potential and chances of malignant potential. A better knowledge of the molecular pathogenesis of ameloblastoma and its various subtypes may provide diagnostic and therapeutic benefits. We are reporting a case of granular cell ameloblastoma arising from the wall of a dentigerous cyst. The lining of the dentigerous cyst shows a potential for neoplastic transformation to ameloblastoma, squamous cell carcinoma, and mucoepidermoid carcinoma.

## Introduction

Odontogenic cysts are developmental or inflammatory in origin, and are derived from the epithelium of the odontogenic apparatus, like from the reduced enamel epithelium, the cell rests of Malassez, and the cell rests of Serres [1]. Reduced enamel epithelium is present over the developing crown of the tooth, cell rests of Malassez are the remnants of Hertwig’s epithelial root sheath, and cell rests of Serres are the remnants of the dental lamina [2,3]. Dentigerous cysts are developmental in origin, occurring around the crown of an unerupted tooth, which may have potential complications. Studies have shown that dentigerous cystic lining may transform into odontogenic tumors and also malignant tumors, such as mucoepidermoid carcinoma [4]. The incidence of the development of neoplasms from odontogenic cysts accounts for <3% [5]. Here, we report a rare case of granular cell ameloblastomatous transformation from the remnants of a dentigerous cyst.

## Case presentation

A 46-year-old female patient reported a swelling on the right side of the lower jaw for one year. The swelling was initially small in size, which gradually increased to the present size. Past dental history revealed the extraction of the third molar tooth. A surgical history of dentigerous cyst enucleation around one and a half years back at the same site, along with the extraction of the associated tooth, was noted according to the dental records. General physical examination did not reveal any abnormality.

**Figure 1:**
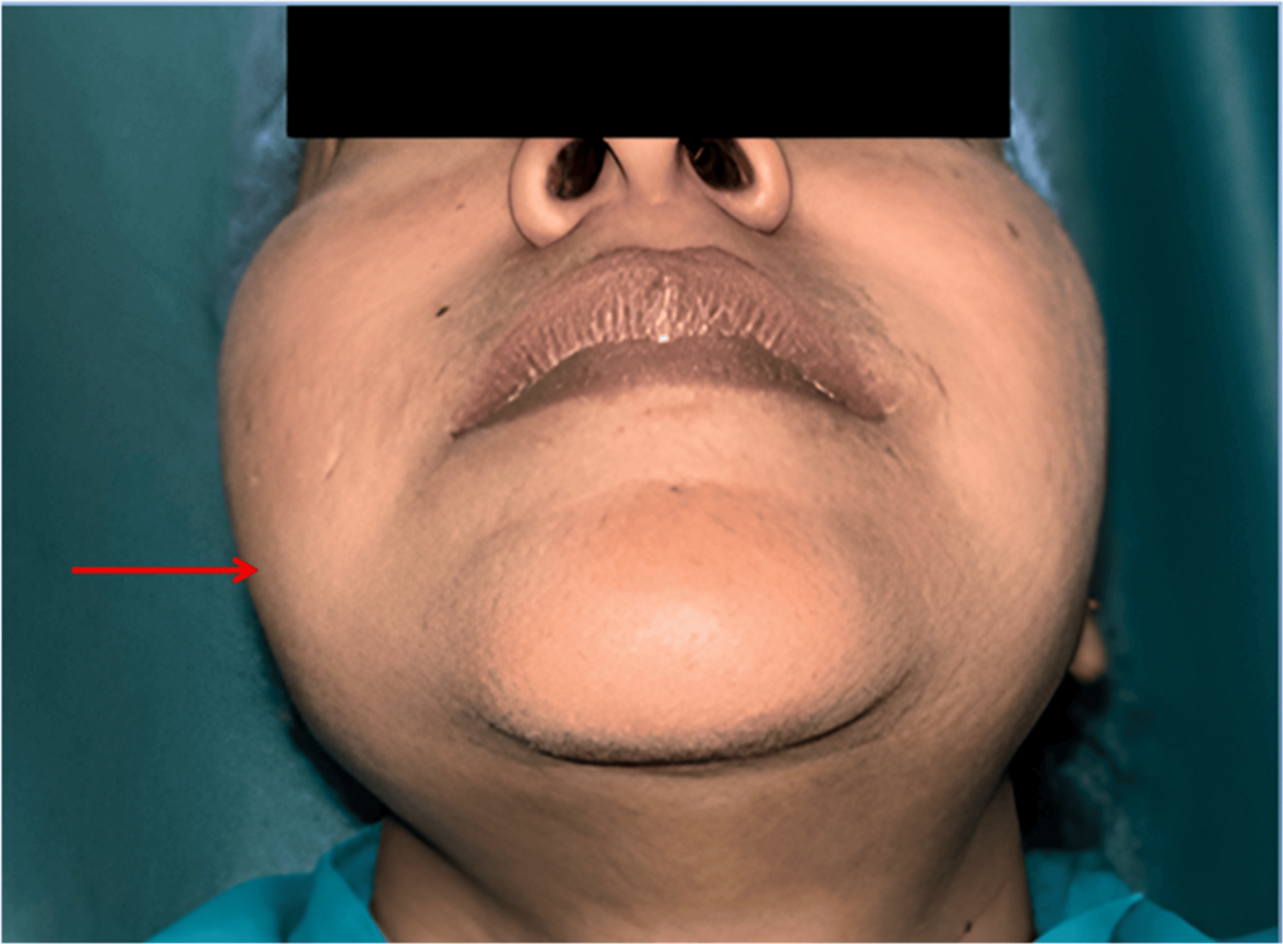
Extra-oral examination showing facial asymmetry with a diffuse swelling on the right side of the face.

Extra-oral examination revealed facial asymmetry with a diffuse swelling on the right side of the face. The swelling extended from the ala-tragus line to 2 cm above the lower border of the mandible and measured around 4 x 4 cm in dimensions (Figure 1). The swelling was firm in consistency and tender on palpation. The right submandibular lymph node was firm and tender on palpation.

**Figure 2:**
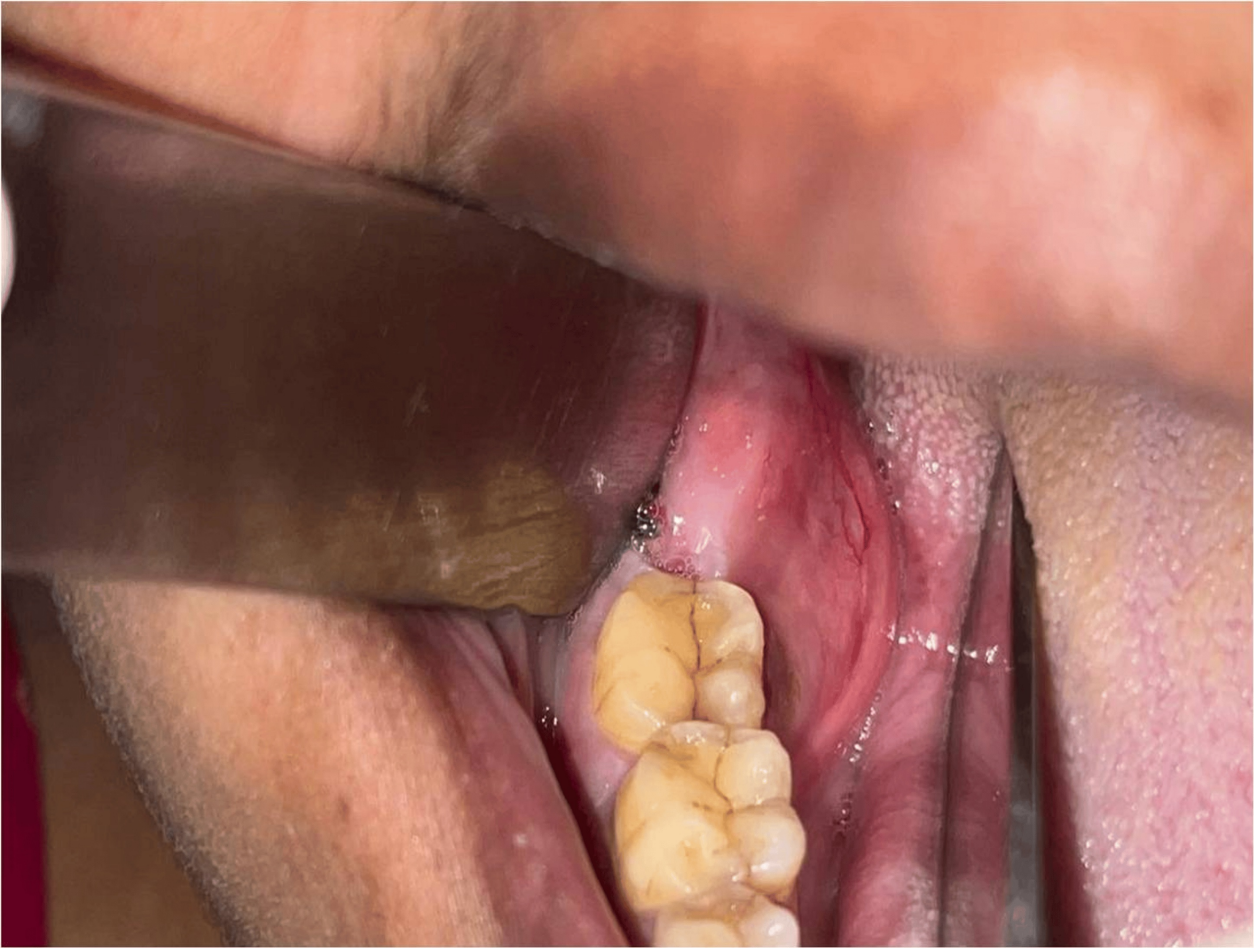
Intra-oral swelling in the right mandibular posterior region.

Intraoral examination revealed a soft, fluctuant, well-defined swelling, obliterating the lingual vestibule with a smooth surface and extending anteroposteriorly from the alveolar ridge of teeth 46 and 47 regions up to the ascending ramus till the occlusal level of upper molars. Upon palpation, the swelling was tender and soft in consistency. Missing teeth were 28, 38, and 48. Deep occlusal caries was seen in tooth 36 (Figure 2).

**Figure 3:**
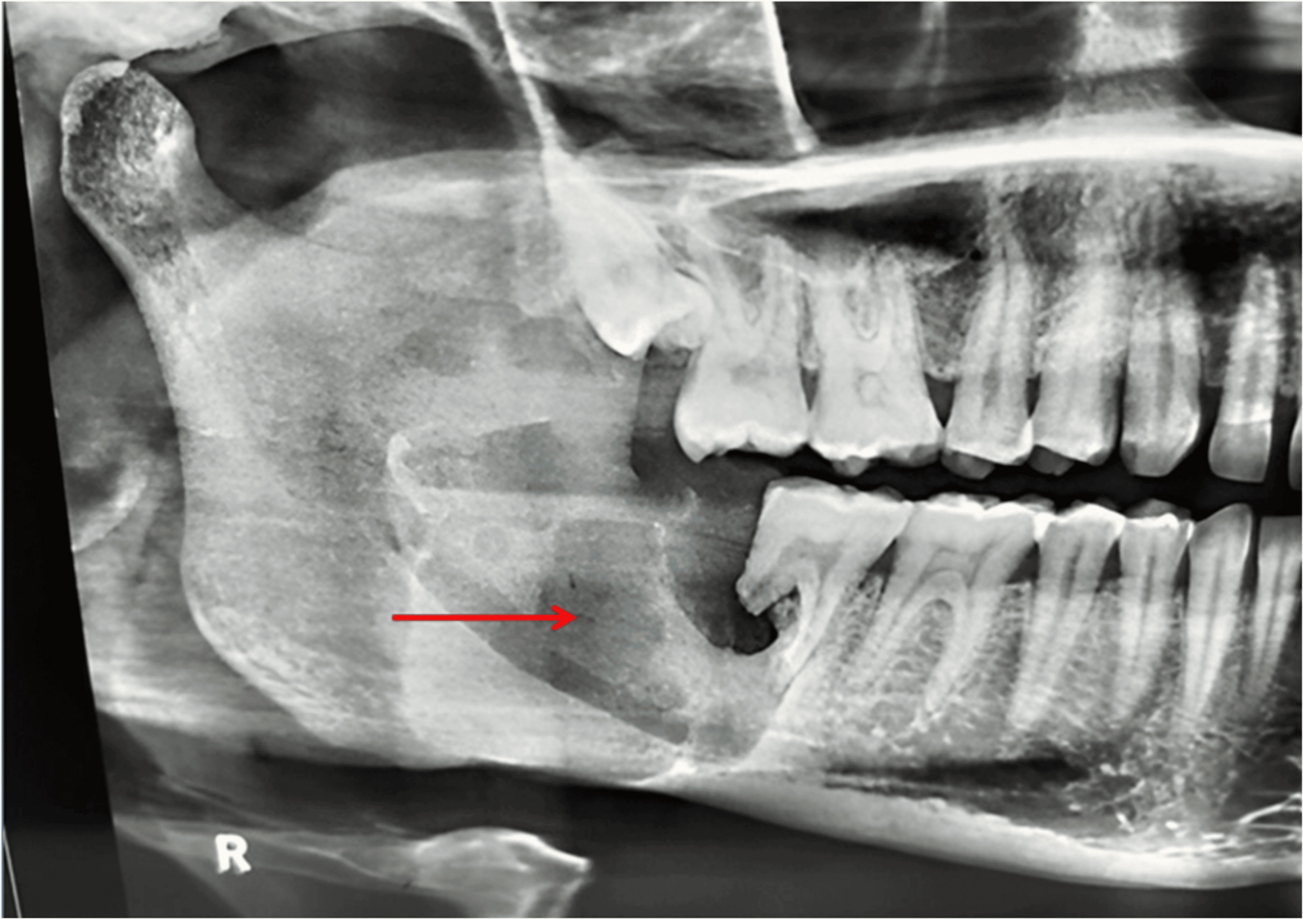
Orthopantomogram revealed unilocular, non-corticated radiolucency on the right side extending from the tooth 47 region to the mid-ramus region.

On radiological examination, orthopantomogram (OPG) revealed unilocular, non-corticated radiolucency on the right side extending from the tooth 47 region till the mid-ramus region. The internal structure was homogenously radiolucent. Buccal as well as lingual cortical perforation with root resorption of tooth with respect to tooth 47 was noted (Figure 3).

**Figure 4:**
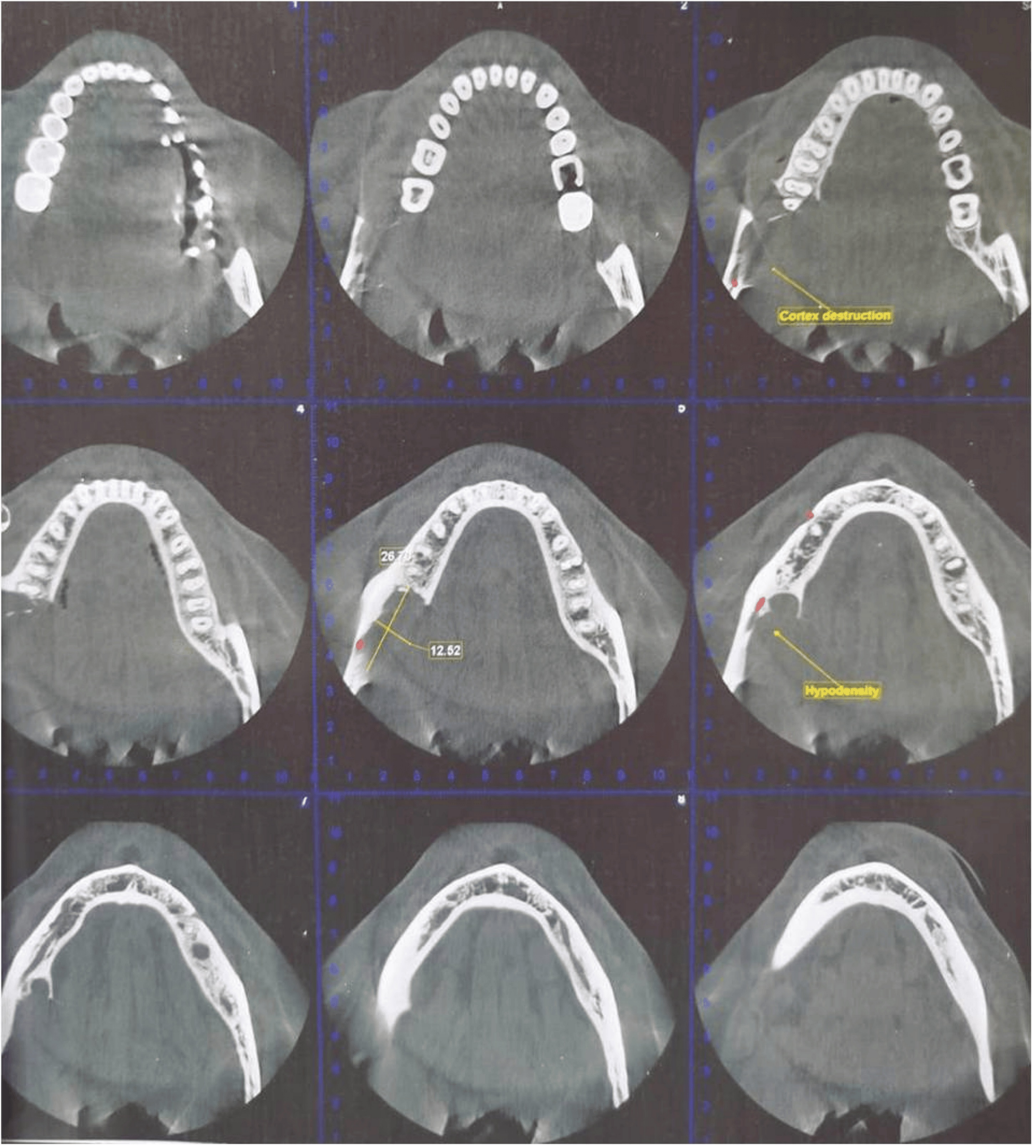
Cone beam computed tomography showing destruction of the buccal and lingual cortex.

Cone beam computed tomography (CBCT) revealed buccal and lingual cortex destruction with inferior alveolar nerve canal involvement and the hypodensity measuring about 12.52 x 26.7 mm in dimensions (axial section) (Figure 4).

On the basis of clinical and radiographic findings, a provisional diagnosis of ameloblastoma was made (as there was a history of extraction of the impacted third molar), with a differential diagnosis of odontogenic keratocyst. The excisional biopsy specimen was sent for histopathological examination.

**Figure 5:**
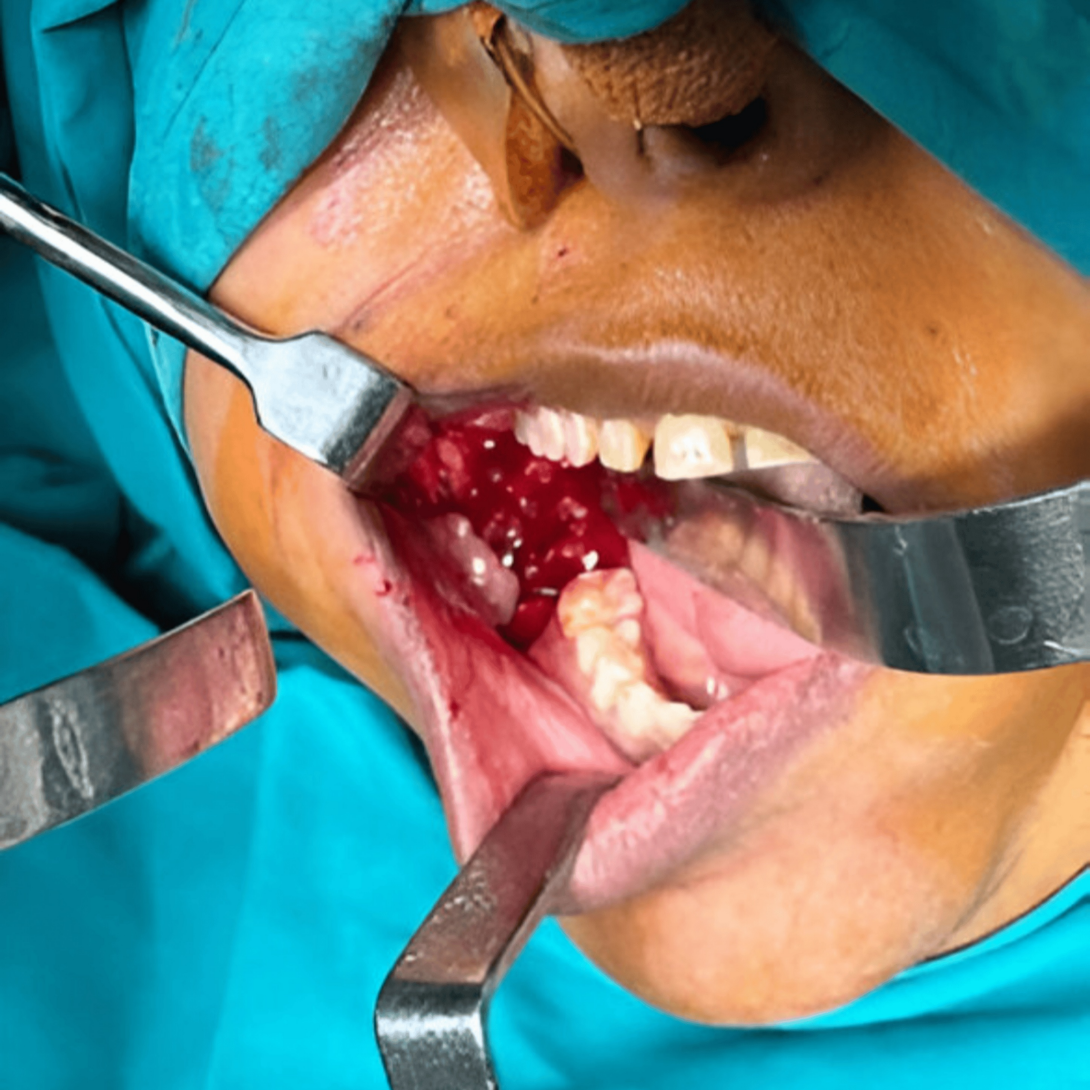
Enucleation of the lesion.

Under general anesthesia, enucleation of the lesion was done along with the removal of the mandibular right second molar. The lesion was mixed with cystic and solid areas (Figure 5).

**Figure 6:**
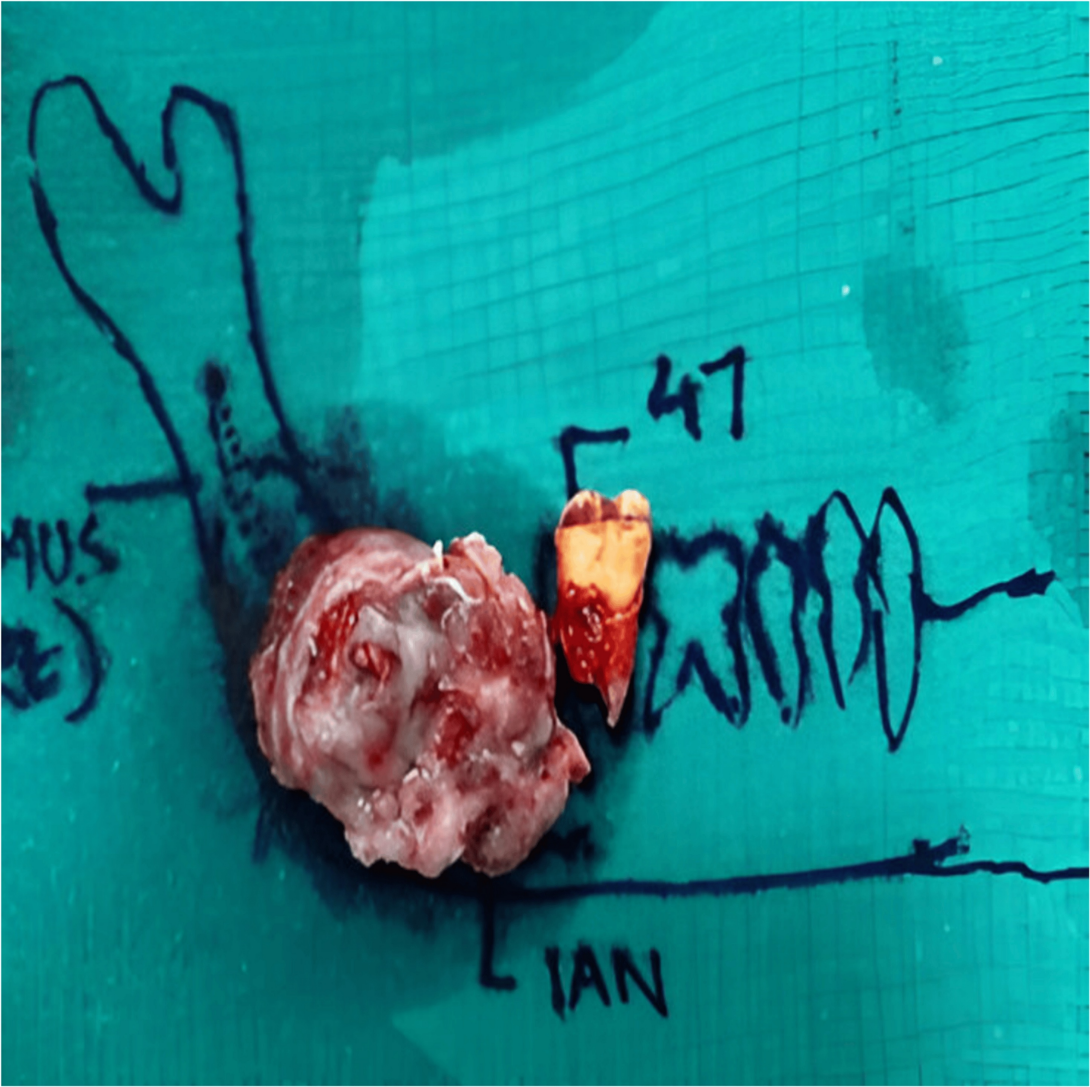
Macroscopic examination revealed a specimen associated with the permanent mandibular right second molar.

Macroscopic examination revealed a specimen showing a swelling of pinkish red in color, measuring approximately 6 x 5 cm in dimensions, roughly oval, firm, and showing regular surface and borders associated with the permanent mandibular right second molar (Figure 6).

**Figure 7:**
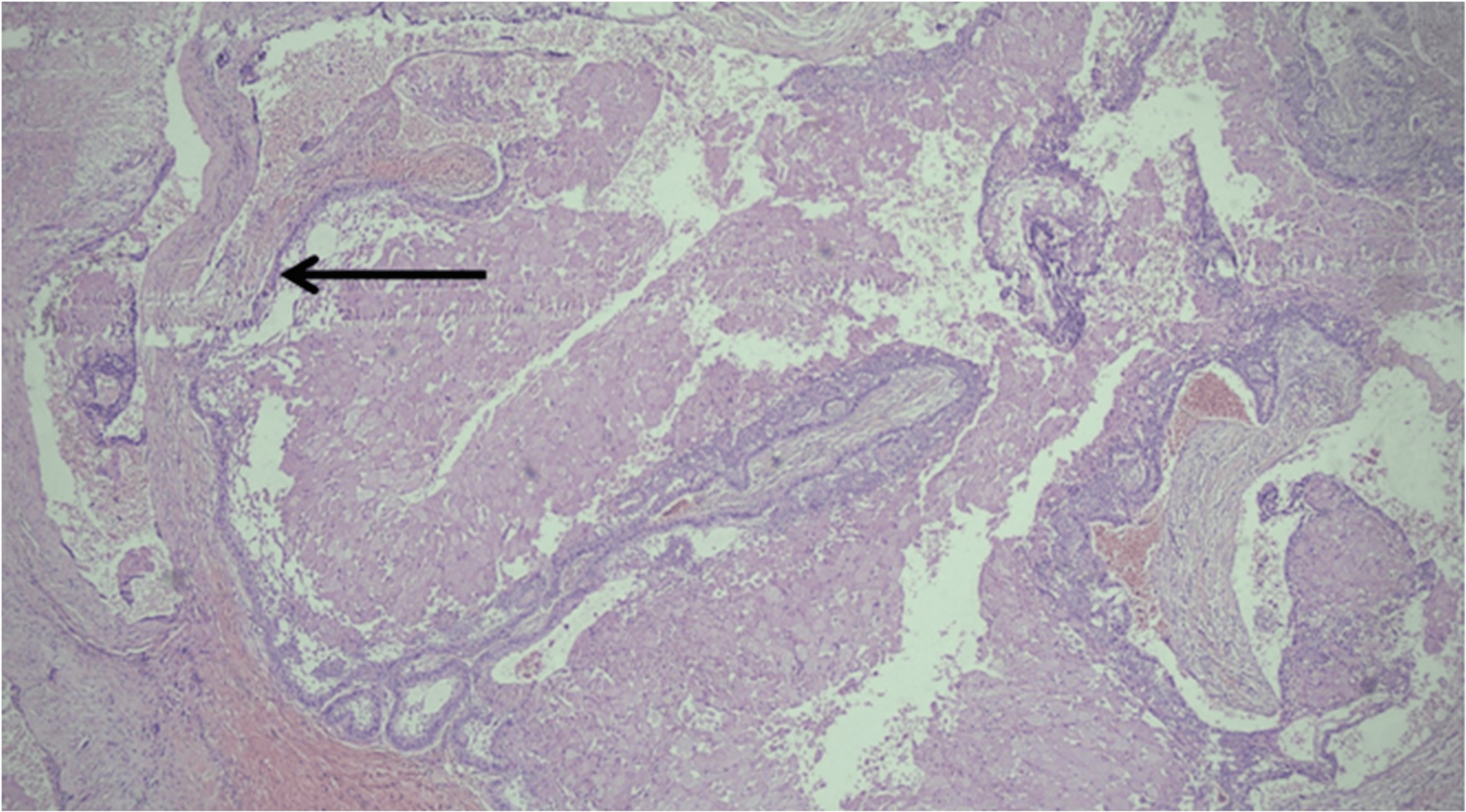
The cystic lining resembling reduced enamel epithelium.

Histopathological examination of one section revealed a well-encapsulated tumor growth with infiltration of the capsule in places. The cystic lining resembled reduced enamel epithelium (Figure 7).

**Figure 8:**
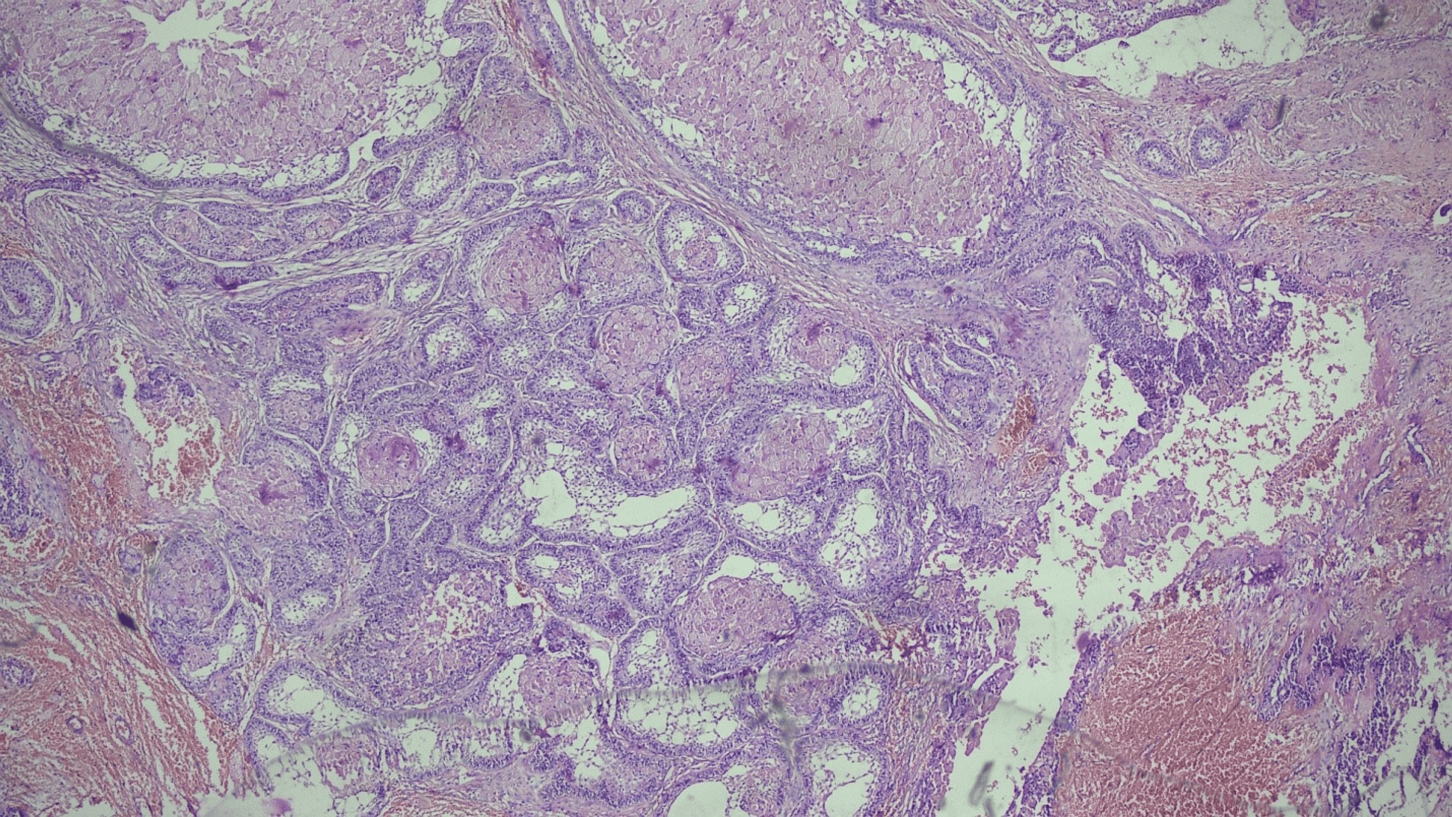
Low-power view showing the tumor consisting of epithelial islands and scant connective tissue.

Low-power view showed a tumor consisting of epithelial islands and scant connective tissue. The islands were lined by tall columnar epithelial cells with nucleus toward the periphery, suggestive of ameloblast-like cells (Figure 8).

**Figure 9:**
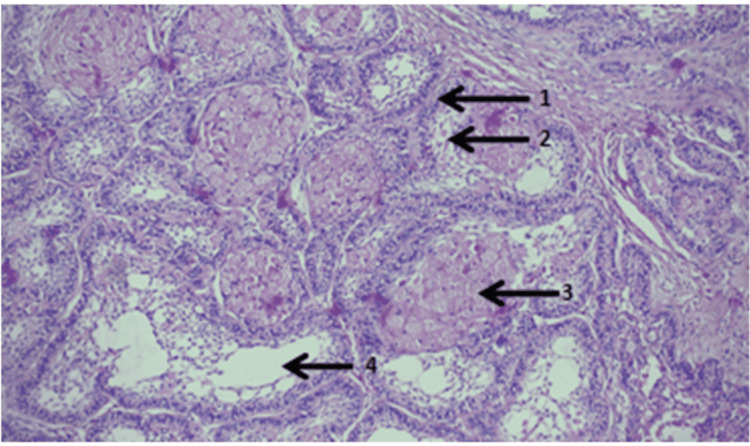
High-power view of islands of tumor cells lined by ameloblast-like cells, and the central area showed granular cells, stellate reticulum-like cells, and areas of cystic degeneration.

High-power view of islands of tumor cells lined by ameloblast-like cells and a central area showed granular cells, stellate reticulum-like cells, and areas of cystic degeneration (Figure 9).

**Figure 10:**
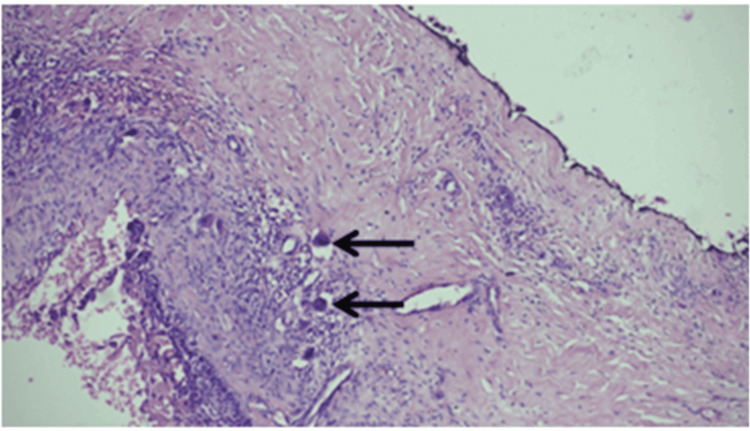
Connective tissue with collagen fibers interspersed with blood elements and a few multinucleated giant cells.

The connective tissue showed collagen fibers interspersed with blood elements, and a few multinucleated giant cells were observed (Figure 10).

Based on these features, a diagnosis of a dentigerous cyst undergoing granular cell ameloblastomatous transformation was given.

## Discussion

Granular cell ameloblastoma is a pathological disease of interest due to its rarity. Its appearance in an edentulous area after dentigerous cyst therapy indicates that the odontogenic epithelium linked to the previously underdiagnosed dentigerous cyst may be aggressive in nature. This abrupt shift draws attention to the intricate character of odontogenic lesions and their potential aggressive behavior.

This tumor is a rare histological type because, unlike other variants of ameloblastomas, the epithelium is hardly recognizable amongst the sheets of granular cells. Reichart et al. reported a recurrence rate of 33.3% for granular cell ameloblastoma, which was higher than the commonly occurring follicular, plexiform, and acanthomatous variants [6].

Dentigerous cyst, also called a follicular cyst, is the second most common odontogenic cyst occurring in the oral cavity after radicular cyst [7]. A very few cases of granular cell ameloblastoma arising from a dentigerous cyst have been reported to date [8]. This transformation is rare and accounts for around 3-5% of cases. Clinically or radiographically, this granular cell ameloblastoma appears like any other ameloblastoma, and the unique features are appreciated only in histopathology [9]. This case report describes a rare case of ameloblastomatous transformation from the dentigerous cyst lining.

Etiological factors proposed for ameloblastomas transformation from odontogenic cysts include extraction, infection, trauma, inflammation, unerupted tooth, nonspecific irritational factors, viral infections, nutritional deficiencies, etc. Ameloblastoma is the most common odontogenic tumor, which accounts for 10% of all tumors [10].

In the present case, there was a history of extraction of an impacted third molar with cystic enucleation one and a half years back.

Dentigerous cysts are believed to be generally associated with impacted or unerupted teeth and arise from the accumulation of fluid between the reduced enamel epithelium and the enamel surface of the crown, which results in an expansion of the follicle beyond its normal dimensions of 3 mm [10].

Ameloblastomas may originate from the remnants of the dental lamina and enamel organ. They may also arise from the basal cell layer of oral epithelium. Radiographic appearance shows unilocular or multilocular radiolucency [11].

The transformation of odontogenic cysts to tumors is indicated by certain features: (1) delay in healing after cystectomy, pain, and swelling; (2) radiographically, malignant changes are generally not detected at the early stage of the disease [12]. At the later stages, unilocular radiolucency is noted with irregular, poorly defined borders and erosion of cortical bone, which indicates an invasive behavior [12].

Sulistyani et al., in their systematic review, discussed that neoplastic lesions may develop from the remnants of odontogenic cysts [13]. Increased intracystic pressure, persistent inflammation, and incomplete removal of cystic lining are associated with this transformation. Chronic inflammation may cause changes at the cellular and genetic levels due to reactive oxygen species such as hydrogen peroxide, superoxide ions, and hydroxyl ions. These are produced by the cells and react with nitric oxide to form reactive nitrogen ions. Reactive nitrogen intermediates may activate carcinogenesis by damaging DNA, proteins, and cell membranes. Chronic inflammation may also induce cell apoptosis, cytokine production, and keratinization of the cystic epithelium, and cause DNA, protein, and cell membrane aberration; therefore, stimulating the transformation of normal cells to neoplastic cells. Odontogenic cysts have the potential for neoplastic transformation, such as ameloblastomas, adenomatoid odontogenic tumors, and even non-odontogenic tumors. This transformation to neoplastic lesions accounts for less than 3%. A study by Borrás-Ferreres et al. reported that neoplastic transformation from a dentigerous cyst may occur without chronic inflammation, suggesting the presence of other physiopathological mechanisms that may be associated with oncogenes [12]. The exact causes of these transformations are not yet known [13].

Granular cell changes in ameloblastoma are an uncommon histopathological feature. This was noted by Krompecher in 1918 and was identified as pseudoxanthomatous cells. The diagnosis of this tumor is confirmed by the presence of granular cells, which gradually replace the stellate reticulum cells. Initially, they were thought to be a degenerative change [14]. Granular cells show an epithelial origin. Ultrastructurally and histochemically, the granular cells are found to be lysosomes. The lysosomal aggregation in the cytoplasm occurs by dysfunction of either (a) lysosomal enzyme or (b) lysosomal-associated protein involved in enzyme activation, targeting, or lysosomal generation [15].

Granular cells showed a high expression of β-catenin and Wnt-5a, mild expression of cone morphogenetic protein-4, and no expression of Wnt-2 [16]. Immunohistochemical studies are conducted on bone morphogenic proteins and Wnt signaling pathway molecules responsible for cell proliferation, cytodifferentiation, and secretion in granular cell ameloblastomas. Normally, the cells synthesize signaling molecules such as β-catenin and Wnt-5a, but their transportation or secretion process is impaired. Therefore, the molecules stored in the cytoplasm act as autophagosomes. The processes of synthesis and secretion of BMP-4 and Wnt-2 are hampered, and the molecules are therefore not stored in the granular cells [17].

Immunohistochemical studies have also proved that the granular cells are positive for cytokeratin, lysozyme, CD68, and alpha-1-antichymotrypsin and negative for desmin, vimentin, neuron-specific enolase, CD15, and S-100 protein, which indicates that they are of epithelial origin and show lysosomal aggregation. Granular cell ameloblastoma, which is treated by enucleation or curettage, exhibits a higher recurrence rate. This may be due to the fact that the tumor border within cancellous bone extends beyond the apparent macroscopic surface and the radiographic periphery of the tumor. Hence radical surgical method is the treatment of choice [18,19].

The differential diagnosis of granular cell ameloblastoma includes oral lesions with granular cell accumulation, including granular cell tumor, granular cell odontogenic tumor, and congenital epulis. Biologic behavior, treatment, and prognosis of these lesions are different and therefore discrimination is of importance [20].

It has been observed that the transformation of odontogenic cysts is slightly more pronounced in males, regardless of the patient's age [21]. In our case, the patient was a 46-year-old female. This underscores the critical importance of prompt diagnosis, treatment, and follow-up. A histological examination is essential ("sine qua non") for the diagnosis of cystic and neoplastic lesions. A thorough histopathological assessment ensures that patients receive appropriate therapy [21].

Radiological "red flags" denote erosion of bone, large size, and involvement of the inferior alveolar nerve, which are commonly associated with neoplasms arising from cystic lesions [22]. Our case exhibited cortical bony perforation and involvement of the inferior alveolar nerve. The treatment of odontogenic cysts to prevent neoplastic transformation remains unstandardized [23]. Several reports suggest that odontogenic cysts should be treated conservatively, with adequate margins. In the case of dentigerous cysts, it is important to remove the impacted tooth immediately so as to prevent the neoplastic transformation of the cystic epithelium remnants [24].

The potential consequences of dentigerous cysts include (a) bone destruction, (b) root resorption, and (c), if untreated, transformation into odontogenic tumors (e.g., ameloblastoma) or malignant tumors (e.g., squamous cell carcinoma) [25]. In cases of large cysts, marsupialization is suggested to prevent fractures or damage to vital organs and tissues during enucleation. However, the majority of the literature suggests that cyst enucleation is the preferred choice of treatment, as marsupialization carries the risk of cystic cell retention and potential conversion into neoplasms [26].

## Conclusions

To ensure an accurate diagnosis and optimal treatment plan, clinicians and pathologists must assess whether a dentigerous cyst is undergoing ameloblastomatous changes. Future advancements in the diagnosis of odontogenic cystic transformation to tumors may benefit from the use of specific immunological markers for neoplastic ameloblastic epithelium. Furthermore, differentiating granular cell ameloblastoma from other granular cell lesions is crucial, as it can guide better treatment strategies and improve patient care.

## References

[1] Rajendra Santosh AB (2020). Odontogenic cysts. Dent Clin North Am.

[2] Brown SJ, Conn BI (2022). Odontogenic cysts: classification, histological features and a practical approach to common diagnostic problems. Diagn Histopathol.

[3] Chi AC, Neville BW (2011). Odontogenic cysts and tumors. Surg Pathol Clin.

[4] Kondamari SK, Taneeru S, Guttikonda VR, Masabattula GK (2018). Ameloblastoma arising in the wall of dentigerous cyst: report of a rare entity. J Oral Maxillofac Pathol.

[5] Wolk DR, Freedman DP, Reich DR (2022). Primary intraosseous squamous cell carcinoma arising in odontogenic cysts: a report of five cases and a review of the literature. Oral Surg Oral Med Oral Pathol Oral Radiol.

[6] Reichart PA, Philipsen HP, Sonner S (1995). Ameloblastoma: biological profile of 3677 cases. Eur J Cancer B Oral Oncol.

[7] Bhushan NS, Rao NM, Navatha M, Kumar BK (2014). Ameloblastoma arising from a dentigerous cyst-a case report. J Clin Diagn Res.

[8] Kahn MA (1989). Ameloblastoma in young persons: a clinicopathologic analysis and etiologic investigation. Oral Surg Oral Med Oral Pathol.

[9] Banerjee A,  Wati SM,  Das M (2024). Aggressive granular cell ameloblastoma arising from radicular cyst: a case report of an unusual variant and a public health concern. J Public Health Emerg.

[10] Leider AS, Eversole LR, Barkin ME (1985). Cystic ameloblastoma: a clinicopathologic analysis. Oral Surg Oral Med Oral Pathol.

[11] Kim SG, Jang HS (2001). Ameloblastoma: a clinical, radiographic, and histopathologic analysis of 71 cases. Oral Surg Oral Med Oral Pathol Oral Radiol Endod.

[12] Borrás-Ferreres J, Sánchez-Torres A, Gay-Escoda C (2016). Malignant changes developing from odontogenic cysts: a systematic review. J Clin Exp Dent.

[13] Sulistyani LD, Iskandar L, Zairinal VN, Arlen AK, Purba F, Ariawan D (2023). Transformation of odontogenic cysts to neoplasms - a systematic review. Ann Maxillofac Surg.

[14] Biradar VG, Latturiya RG, Biradar SV (2012). Granular cell ameloblastoma: a diagnostic dilemma for histopathologist. Eur J Gen Dent.

[15] Martin Y, Sathyakumar M, Premkumar J, Magesh KT (2017). Granular cell ameloblastoma. J Oral Maxillofac Pathol.

[16] Sathi GS, Han PP, Tamamura R, Nagatsuka H, Hu H, Katase N, Nagai N (2007). Immunolocalization of cell signaling molecules in the granular cell ameloblastoma. J Oral Pathol Med.

[17] Yamunadevi A, Madhushankari GS, Selvamani M, Basandi PS, Yoithapprabhunath TR, Ganapathy N (2014). Granularity in granular cell ameloblastoma. J Pharm Bioallied Sci.

[18] Dina R, Marchetti C, Vallania G, Corinaldesi G, Eusebi V (1996). Granular cell ameloblastoma: an immunocytochemical study. Pathol Res Pract.

[19] Nikitakis NG, Tzerbos F, Triantafyllou K, Papadimas C, Sklavounou A (2011). Granular cell ameloblastoma: an unusual histological subtype report and review of literature. J Oral Maxillofac Res.

[20] Sukumaran R, Somanathan T, Sen A (2024). Granular cell ameloblastoma: a rare and unique variant. Indian J Case Rep.

[21] Prasad H, Anuthama K, Chandramohan M, Sri Chinthu KK, Ilayaraja V, Rajmohan M (2015). Squamous cell carcinoma arising from a dentigerous cyst - report of a case and review of literature. J Oral Maxillofac Surg Med Pathol.

[22] Garzino-Demo P, Bianchi CC, Romeo I, Malandrino MC, Cocis S (2020). PIC developing from odontogenic cysts: clinical and radiological considerations on a series of 6 cases. Oral Maxillofac Surg.

[23] Bhuyan L, Nishat R, Behura SS, Mahapatra N, Kumar H (2021). Insight into the molecular pathogenesis of odontogenic lesions. J Oral Biosci.

[24] Mahajan AD, Manjunatha BS, Khurana NM, Shah N (2014). Unicystic ameloblastoma arising from a residual cyst. BMJ Case Rep.

[25] Bilodeau EA, Collins BM (2017). Odontogenic cysts and neoplasms. Surg Pathol Clin.

[26] Bereket C, Bekçioğlu B, Koyuncu M, Şener İ, Kandemir B, Türer A (2013). Intraosseous carcinoma arising from an odontogenic cyst: a case report. Oral Surg Oral Med Oral Pathol Oral Radiol.

